# Supplementary material for: BAX Redistribution Induces Apoptosis Resistance and Selective Stress Sensitivity in Human HCC
Source: Cancers (Basel). 2020 May 31;12(6):1437. doi: 10.3390/cancers12061437 (PMC7352885; doi:10.3390/cancers12061437)
Supplement: Supplementary file 1 [file cancers-12-01437-s001.pdf]

cancers MDPI

Article

BAX Redistribution Induces Apoptosis Resistance and Selective Stress Sensitivity in Human HCC

Kathrin Funk, Carolin Czauderna, Ramona Klesse, Diana Becker, Jovana Hajduk, Aline Oelgeklaus, Frank Reichenbach, Franziska Todt, Joachim Lauterwasser, Peter R. Galle, Frank Edlich and Jens U. Marquardt

Supplementary Materials:

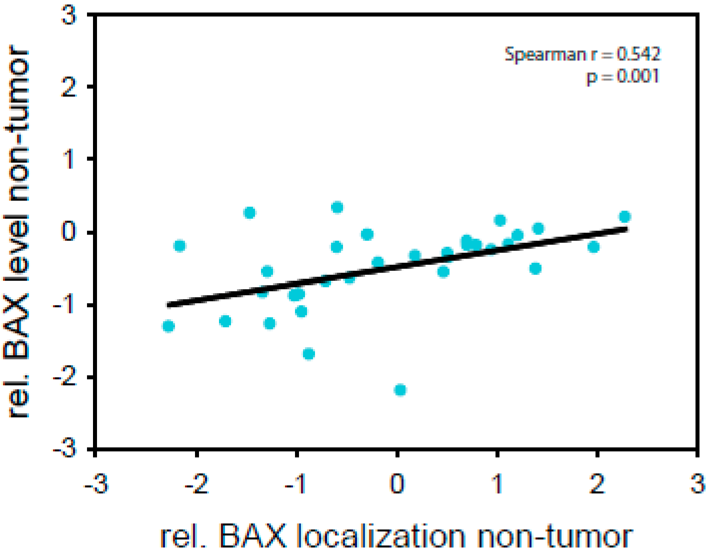

Figure S1. Relative BAX localization in non-tumor samples vs. relative BAX level in non-tumor tissue (double log10 scale) is displayed for 34 patients. Pearson's correlation is shown by r- and *p*-values.

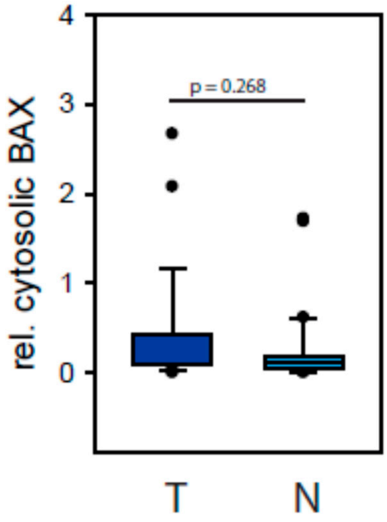

Figure S2. Relative cytosolic BAX (S2) and BAK (S3) levels are displayed for tumor (T) and non-tumor (N) samples from 34 HCC patients. *p*-value according to t-test is displayed.

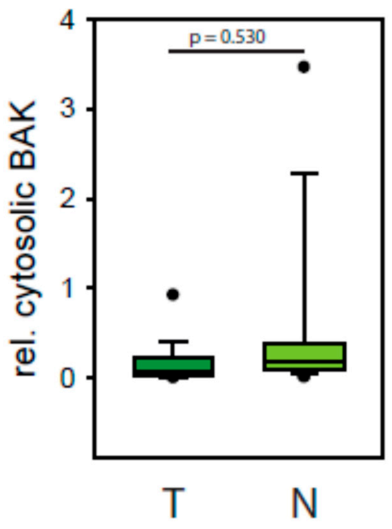

Figure S3. Relative cytosolic BAX (S2) and BAK (S3) levels are displayed for tumor (T) and non-tumor (N) samples from 34 HCC patients. *p*-value according to t-test is displayed.

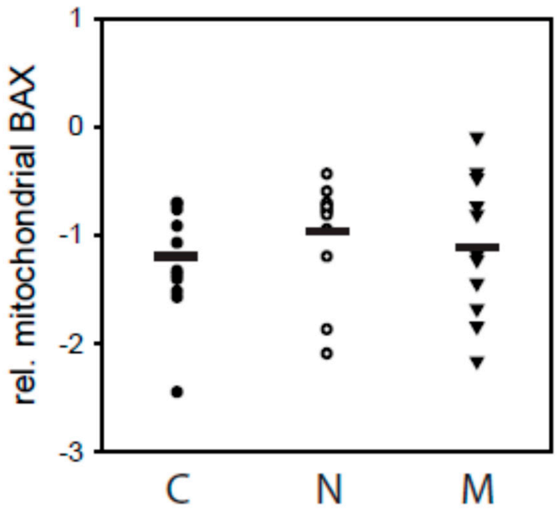

Figure S4. Relative mitochondrial BAX levels in tumor tissues from HCC patients with predominantly cytosolic (C, N = 11), neutral (N, N = 12) and mostly mitochondrial (M, N = 11) BAX localization in non-tumor tissue. Mean is displayed.

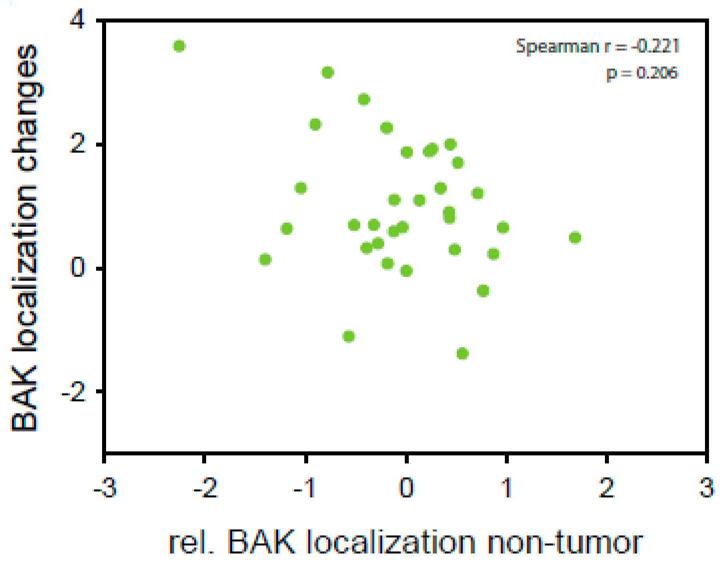

Figure S5. Relative BAK localization in non-tumor tissue vs. changes in relative BAK localization from non-tumor to tumor samples (double log10 scale). r- and *p*-values according Pearson's correlation are displayed.

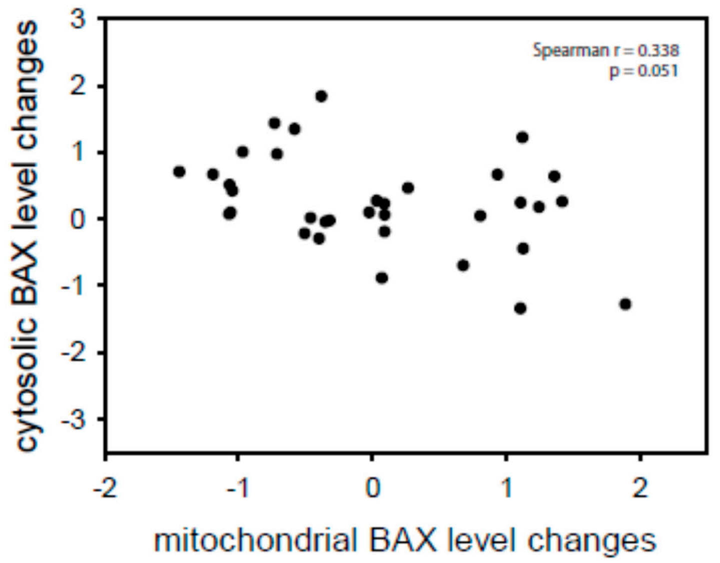

Figure S6. Changes from non-tumor to tumor mitochondrial BAX levels vs. changes from non-tumor to tumor cytosolic BAX levels in 34 HCC patients (double log10 scale). r- and *p*-values according Pearson's correlation are displayed.

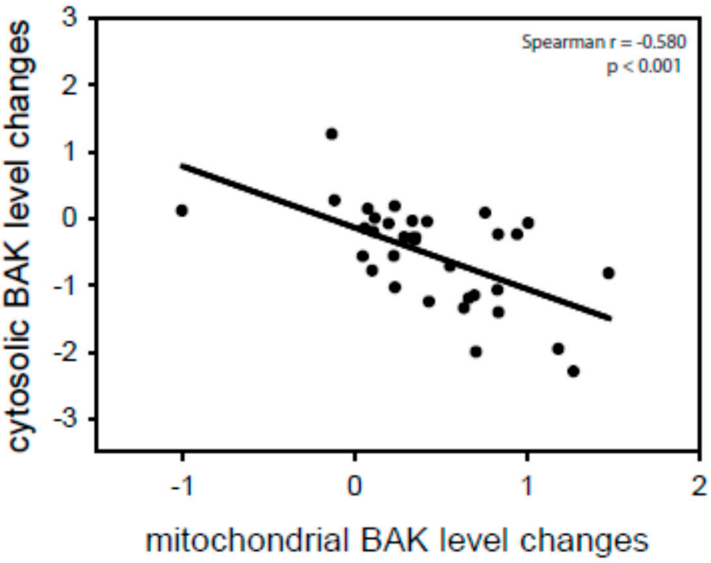

Figure S7. Changes between non-tumor and tumor mitochondrial BAK levels vs. changes between non-tumor and tumor cytosolic BAK levels (double log10 scale). r- and *p*-values according Pearson's correlation are displayed.

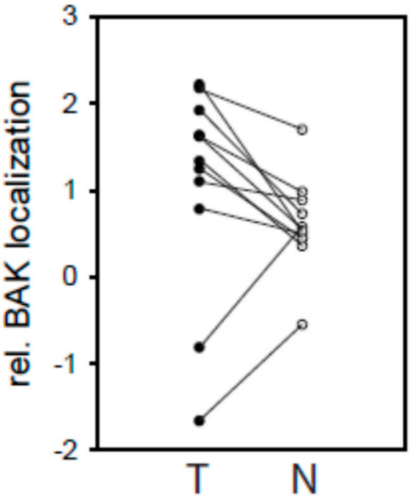

Figure S8. Relative mitochondrial BAK levels are displayed for tumor (T) and non-tumor (N) samples from HCC patients of the protected group (N = 11).

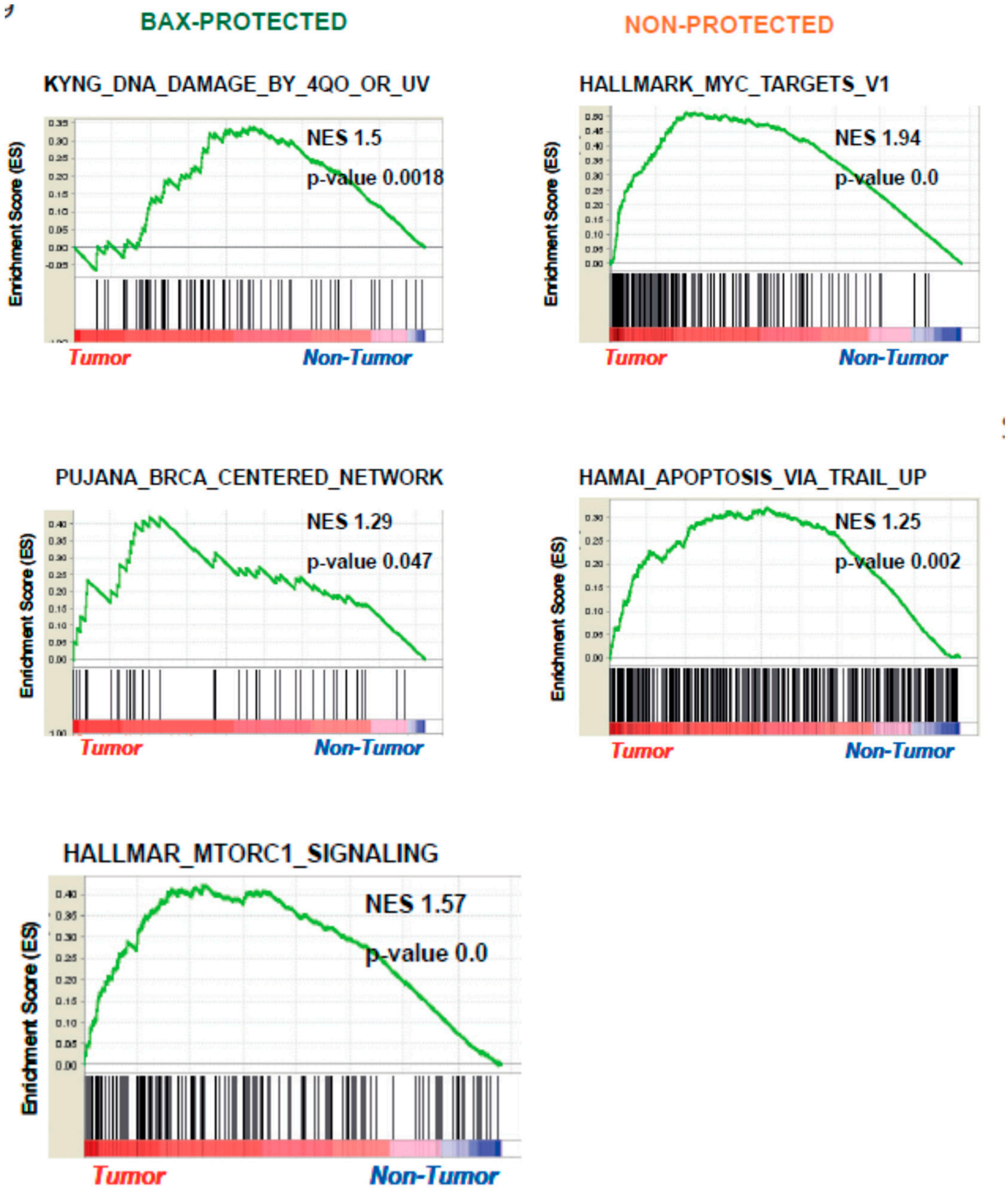

Figure S9. Gene set enrichment analysis (GSEA) for protection and non-protection associated gene expression signatures during malignant transformation were performed. Normalized enrichment score (NES) reflects degree of overrepresentation for each group at the peak of the entire set. Statistical significance calculated by nominal *p*-value of the ES by using an empirical phenotype-based permutation test.

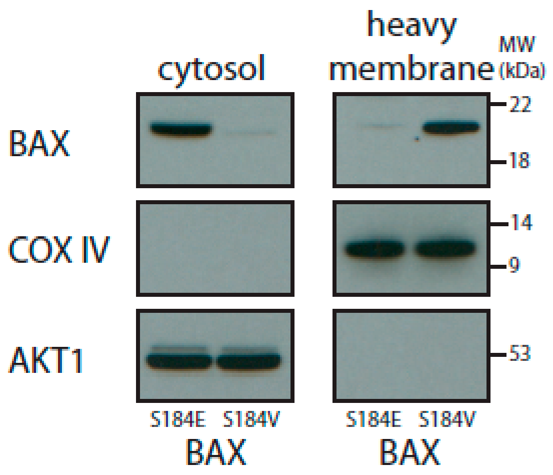

Figure S10. Subcellular localization of BAX S148E and BAX S184V expressed for 4 h in HCT116 BAX/BAK DKO cells after fractionation analyzed by Western blot. Separation of cytosol and heavy membrane fraction (mitochondria) was controlled using AKT1 and COX IV, respectively. N = 3.

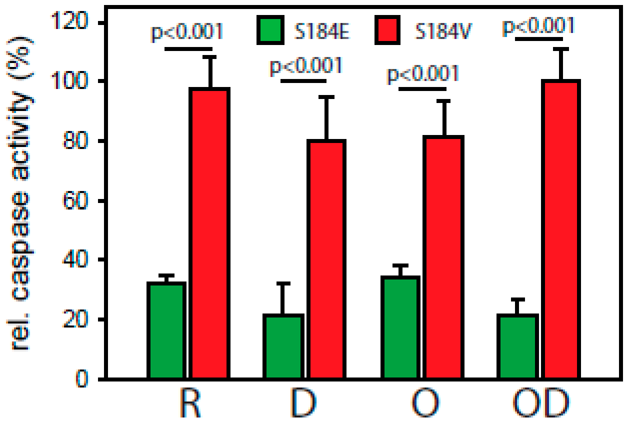

Figure S11. Caspase 3/7 activity induced by 1 μM Rapamycin in HCT116 BAX/BAK DKO cells expressing either the largely cytosolic BAX S184E (green) or the predominantly mitochondrial BAX S184V (red) for 4 h. Data ± SEM. N = 3 and *p*-values according to One Way ANOVA using the Holm-Sidak method are displayed. Data was adjusted to BAX variant expression levels based on GFP fluorescence.

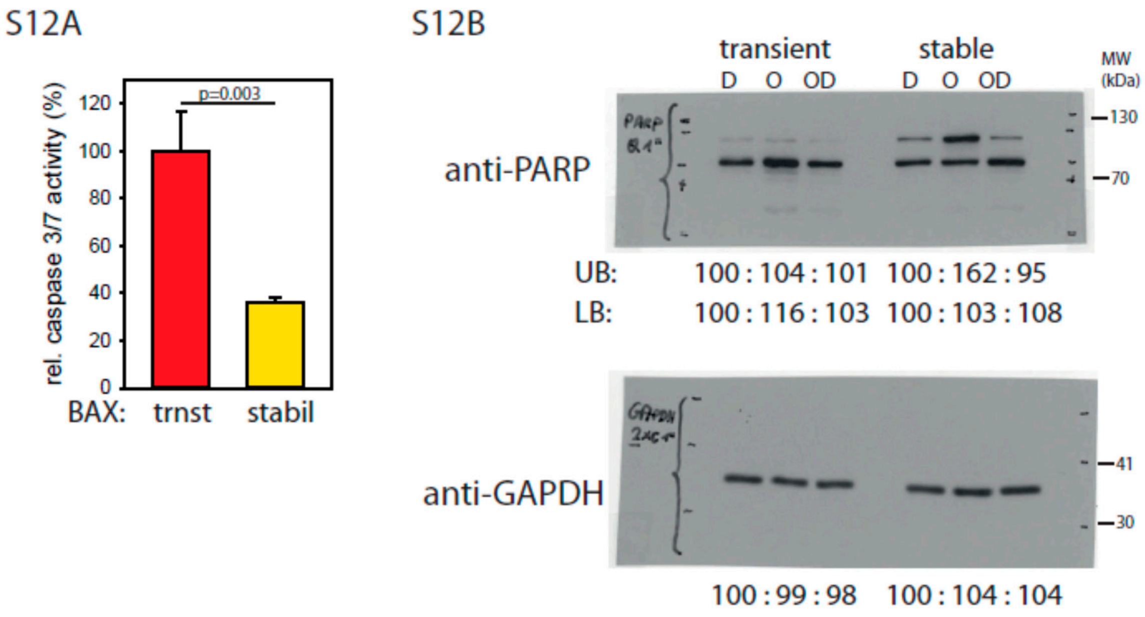

Figure S12. (**A**) Caspase 3/7 activity induced by 1 μM Rapamycin in HCT116 BAX/BAK DKO cells with either transient (red, trnst) or stable (yellow, stable) expression of predominantly mitochondrial BAX S184V. Data ± SEM. N = 3 and *p*-values according to One Way ANOVA using the Holm-Sidak method are displayed. Data was adjusted to BAX S184V expression based on GFP fluorescence. (**B**)Uncropped Western blot depicted in Figure 3H with density quantifications below (UB—upper band, LB —lower band).

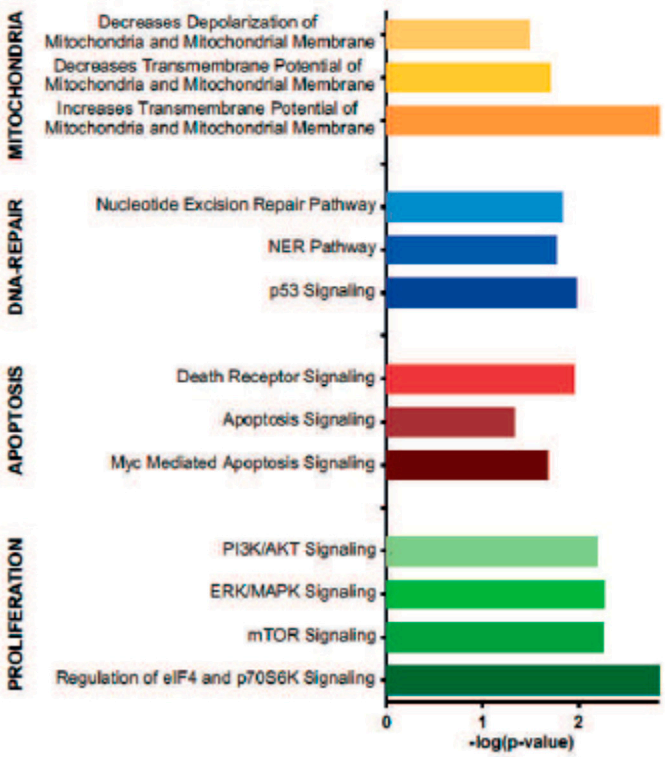

Figure S13. set enrichment analysis (GSEA) for protection and non-protection associated gene expression signatures during malignant transformation were performed. Normalized enrichment score (NES) reflects degree of overrepresentation for each group at the peak of the entire set. Statistical significance calculated by nominal *p*-value of the ES by using an empirical phenotype-based permutation test.

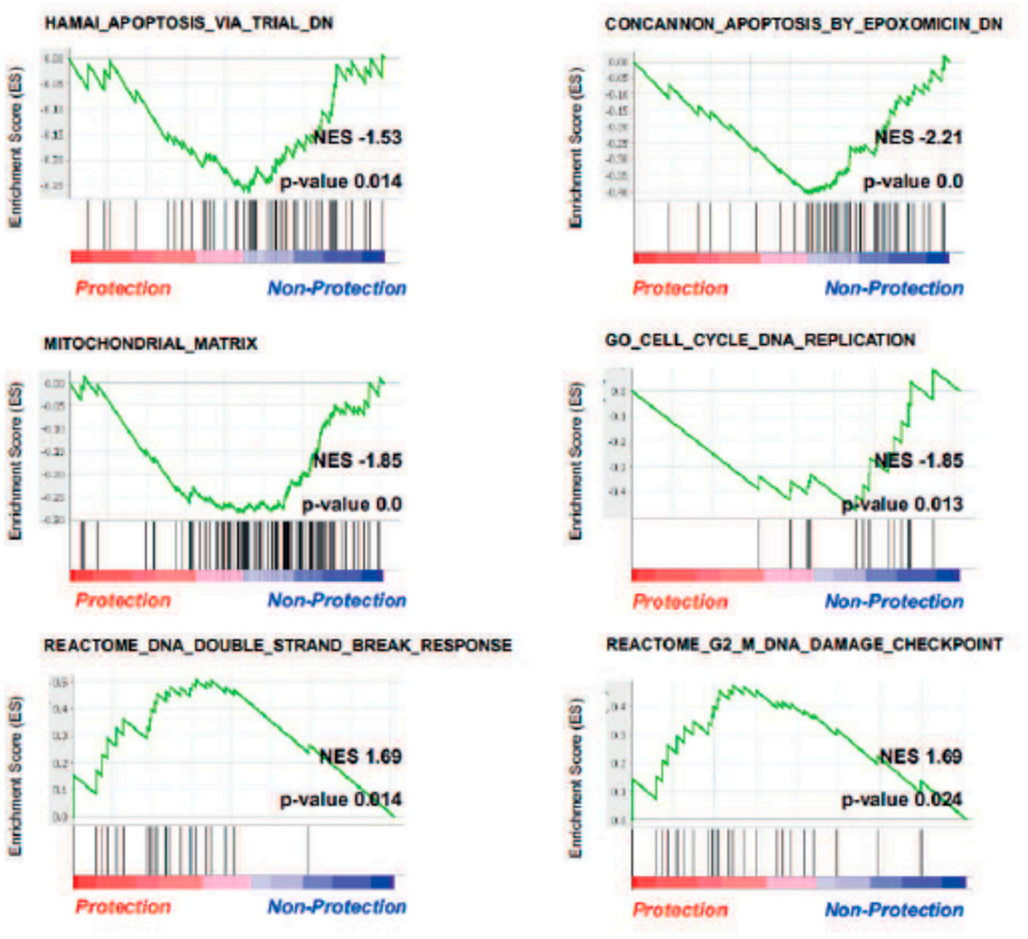

Figure S14. set enrichment analysis (GSEA) for protection and non-protection associated gene expression signatures during malignant transformation were performed. Normalized enrichment score (NES) reflects degree of overrepresentation for each group at the peak of the entire set. Statistical significance calculated by nominal *p*-value of the ES by using an empirical phenotype-based permutation test.

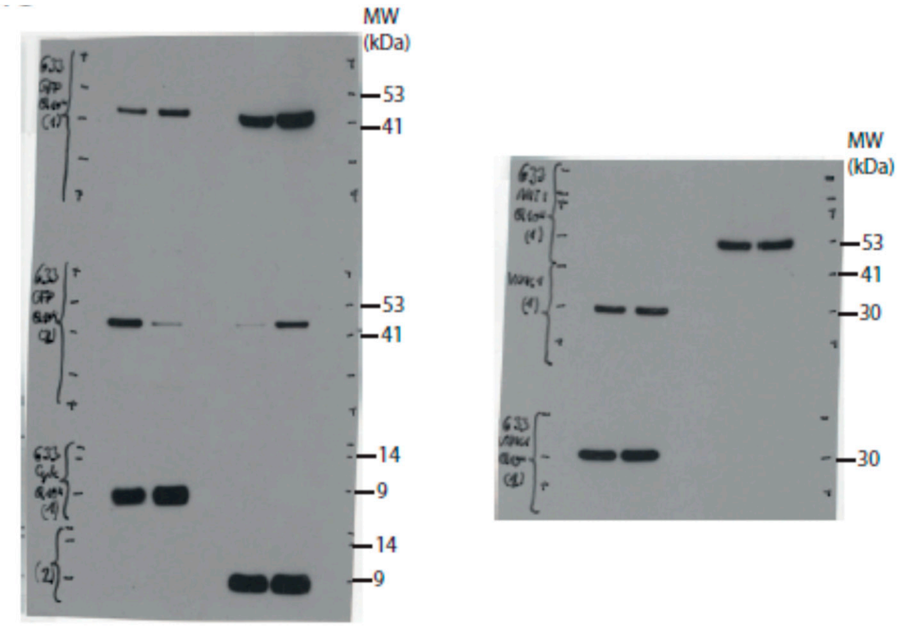

Figure S15. Uncropped Western blot depicted in Figure 4D, F.

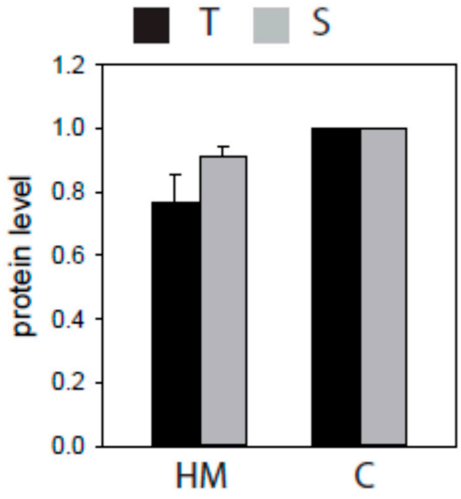

Figure S16. Density quantification of Western blot depicted in Figure 4D.

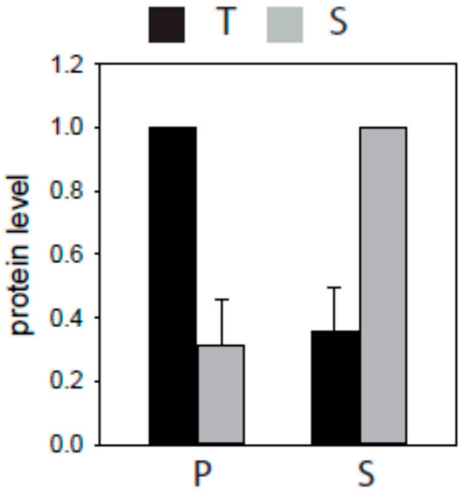

Figure S17. Density quantification of Western blot depicted in Figure 4F.

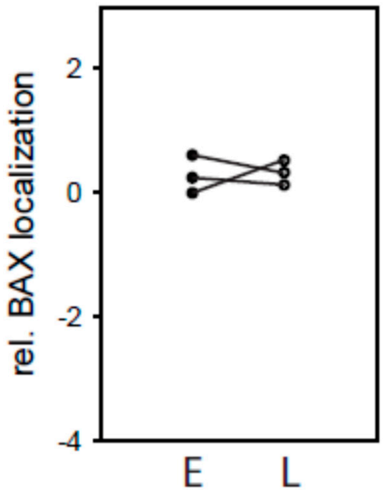

Figure S18. Relative BAX localizations (log10 scale) of polyclonal cell lines (HCC9, HCC31, HCC68) in early (E) and late (L) passages are displayed.

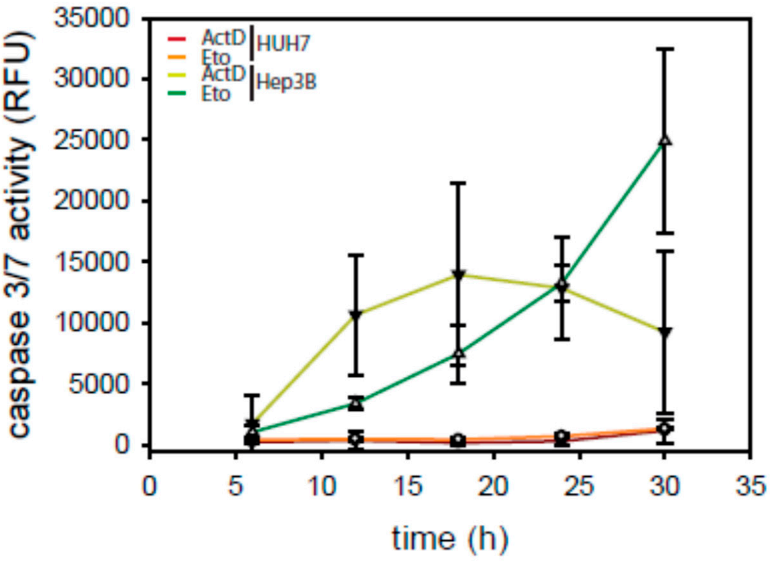

Figure S19. Caspase 3/7 activity of HUH7 cells and Hep3B cells after treatment with 1 μM ActD or 50 μM Etoposide. Data ± SEM. N = 3.

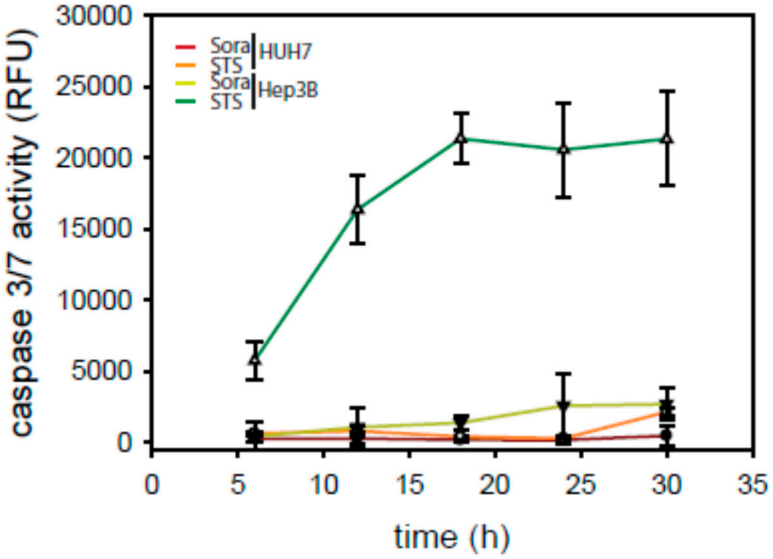

Figure S20. Caspase 3/7 activity measured in HUH7 cells and Hep3B cells in response to 1 μM Staurosporine or 5 μM Sorafenib is displayed. Data ± SEM. N = 3.

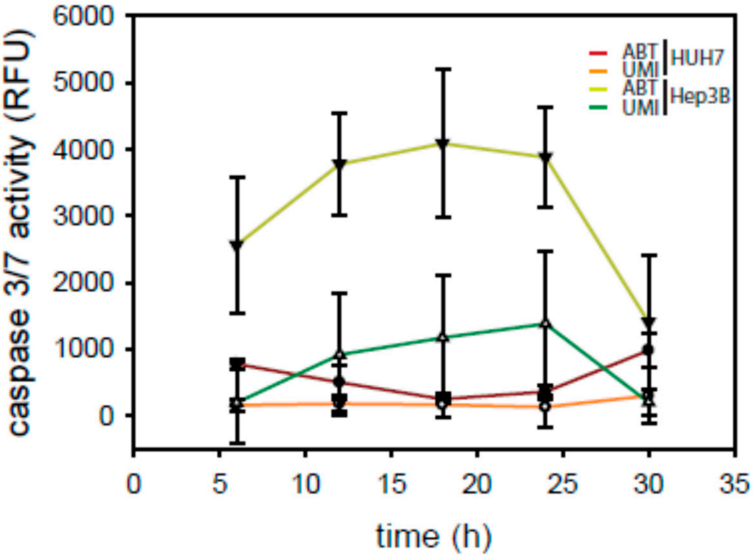

Figure S21. Caspase 3/7 activity resulting from treatment of HUH7 cells and Hep3B cells with 1 µM ABT-737 or 1 µM UMI-77. Data ± SEM. N = 3.

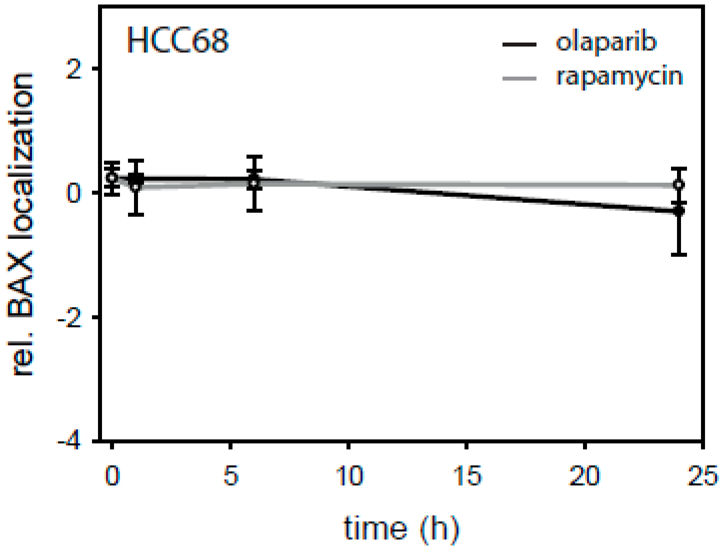

Figure S22. Relative BAX localization of HCC68 cells in the presence of either Olaparib (black) or Rapamycin (gray) monitored at 1, 6 and 24 h. Data represent averages ± SEM. N ≥ 3.

© 2020 by the authors. Submitted for possible open access publication under the terms and conditions of the Creative Commons Attribution (CC BY) license (http://creativecommons.org/licenses/by/4.0/).
